# Open-Source, Step-Counting Algorithm for Smartphone Data Collected in Clinical and Nonclinical Settings: Algorithm Development and Validation Study

**DOI:** 10.2196/47646

**Published:** 2023-11-15

**Authors:** Marcin Straczkiewicz, Nancy L Keating, Embree Thompson, Ursula A Matulonis, Susana M Campos, Alexi A Wright, Jukka-Pekka Onnela

**Affiliations:** 1 Department of Biostatistics Harvard T.H. Chan School of Public Health Boston, MA United States; 2 Department of Health Care Policy Harvard Medical School Boston, MA United States; 3 Division of General Internal Medicine Brigham and Women’s Hospital Boston, MA United States; 4 Dana Farber Cancer Institute Harvard Medical School Boston, MA United States

**Keywords:** accelerometer, cancer, open-source, smartphone, step count, validation, wearable

## Abstract

**Background:**

Step counts are increasingly used in public health and clinical research to assess well-being, lifestyle, and health status. However, estimating step counts using commercial activity trackers has several limitations, including a lack of reproducibility, generalizability, and scalability. Smartphones are a potentially promising alternative, but their step-counting algorithms require robust validation that accounts for temporal sensor body location, individual gait characteristics, and heterogeneous health states.

**Objective:**

Our goal was to evaluate an open-source, step-counting method for smartphones under various measurement conditions against step counts estimated from data collected simultaneously from different body locations (“cross-body” validation), manually ascertained ground truth (“visually assessed” validation), and step counts from a commercial activity tracker (Fitbit Charge 2) in patients with advanced cancer (“commercial wearable” validation).

**Methods:**

We used 8 independent data sets collected in controlled, semicontrolled, and free-living environments with different devices (primarily Android smartphones and wearable accelerometers) carried at typical body locations. A total of 5 data sets (n=103) were used for cross-body validation, 2 data sets (n=107) for visually assessed validation, and 1 data set (n=45) was used for commercial wearable validation. In each scenario, step counts were estimated using a previously published step-counting method for smartphones that uses raw subsecond-level accelerometer data. We calculated the mean bias and limits of agreement (LoA) between step count estimates and validation criteria using Bland-Altman analysis.

**Results:**

In the cross-body validation data sets, participants performed 751.7 (SD 581.2) steps, and the mean bias was –7.2 (LoA –47.6, 33.3) steps, or –0.5%. In the visually assessed validation data sets, the ground truth step count was 367.4 (SD 359.4) steps, while the mean bias was –0.4 (LoA –75.2, 74.3) steps, or 0.1%. In the commercial wearable validation data set, Fitbit devices indicated mean step counts of 1931.2 (SD 2338.4), while the calculated bias was equal to –67.1 (LoA –603.8, 469.7) steps, or a difference of 3.4%.

**Conclusions:**

This study demonstrates that our open-source, step-counting method for smartphone data provides reliable step counts across sensor locations, measurement scenarios, and populations, including healthy adults and patients with cancer.

## Introduction

Walking is the most common form of physical activity [1]. It is also important to prevent chronic disease and premature mortality [2-4]. The recent proliferation and integration of wearable activity trackers into public health and clinical research studies have allowed investigators to identify gait-related biomarkers, such as decreased daily step counts, as risk factors for cardiovascular disease, cancer, stroke, dementia, and type 2 diabetes [5-11].

Despite the potential for wearable activity trackers to increase physical activity, improve health, and provide unique behavioral insights, there are several important limitations. First, the adoption of wearables is uneven across the population, and most people stop using wearable activity trackers after 6 months [12-15]. Second, commercial devices rarely allow access to their raw (subsecond-level) data or provide open-source algorithms for processing data into clinically meaningful end points [16-18]. Third, the accuracy of step count estimates is affected by metrological and behavioral factors, such as the location of the wearable on the body and temporal gait speed [19-21].

Smartphones are a promising alternative for collecting objective, scalable, and reproducible data about human behavior [22-25]. Although smartphones can overcome many limitations of wearable activity trackers (eg, through access to raw sensor data [26] and increased adoption among older individuals [27]), the quantification of gait-related biomarkers remains challenging. This is largely because of the variation in the location and orientation of smartphones in relation to the body in real-life conditions, which affects the data collected from smartphones’ inertial sensors [28-30].

To address this problem, we have recently proposed an open-source walking recognition method [30], which can be applied to accelerometer data collected from various locations on the body, making it suitable for smartphones. In this paper, we demonstrate how our method can be used for quantifying steps, and we validate its performance in 8 independent data sets. We validate this method in three ways: (1) “cross-body validation” compares step counts estimated from multiple sensors worn simultaneously at predesignated body locations; (2) “visually assessed validation” compares step counts estimated from a sensor worn at an unspecified body location against a visually assessed and manually annotated ground truth; and (3) “commercial wearable validation” compares step counts estimated from a sensor worn at an unspecified body location against estimates provided by an independent commercial activity tracker (Fitbit Charge 2) worn on the wrist. The first (“cross-body”) and second (“visually assessed”) validations involve healthy participants whose data were obtained from publicly available data sets collected in controlled, semicontrolled, and free-living conditions, while the third (“commercial wearable”) validation includes data collected by our team from patients with advanced cancer receiving chemotherapy as outpatients in free-living conditions.

## Methods

### Step-Counting Algorithm

**Figure 1:**
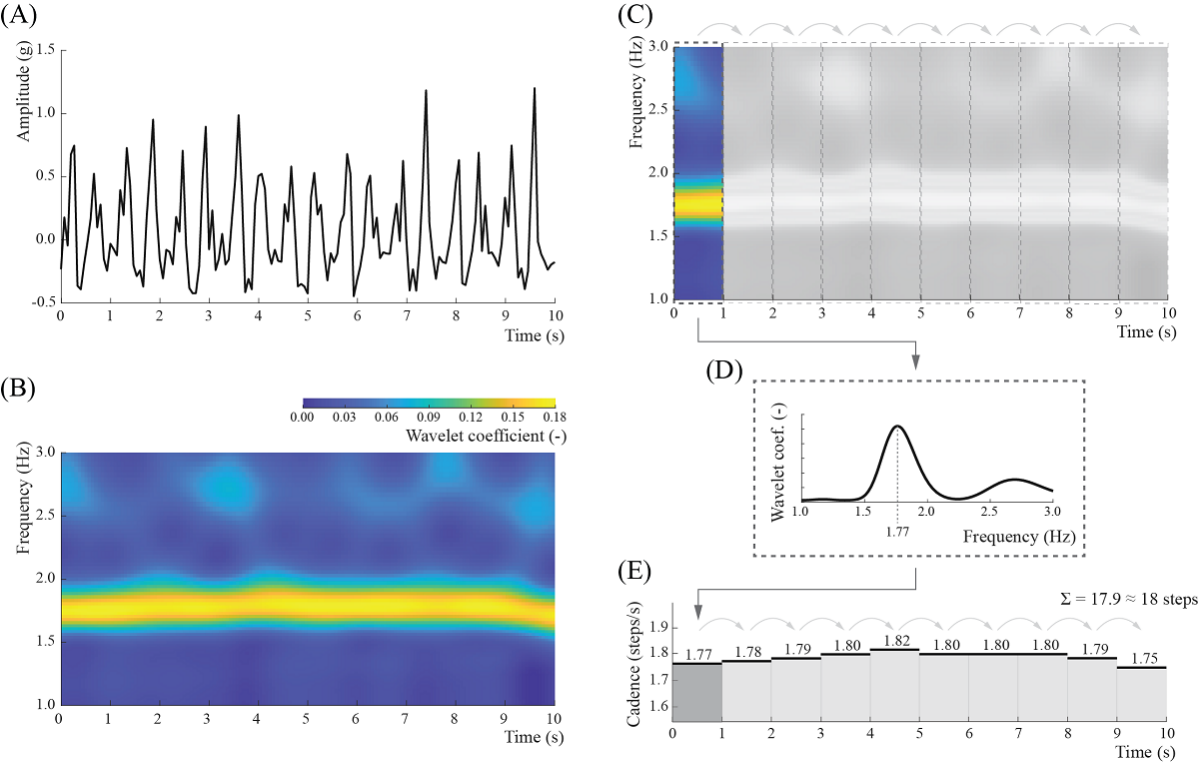
The step-counting algorithm. (A) The original signal is projected onto (B) the time-frequency space using wavelet transformation, which shows the relative weights of different frequencies over time (brighter color indicates higher weight). (C) This scalogram is then split into nonoverlapping 1-second windows. (D) The temporal step frequency (cadence) is estimated as a frequency with the maximum average wavelet coefficient inside each window. (E) The total number of steps in a signal is calculated as a rounded sum of all 1-second counts in that signal.

Our method leveraged the observation that regardless of the sensor location, orientation, or person, during walking, the activity device’s accelerometer signal oscillates around a local mean with a frequency equal to the performed steps [30]. To extract this information, we used the continuous wavelet transform to project the original signal onto the time-frequency space of wavelet coefficients, which are maximized when a particular frequency matches the frequency of the observed signal at a given time point (Figure 1). To translate this information into the number of steps, we split the projection into nonoverlapping 1-second windows, and we estimated the temporal step frequency as a frequency with the maximum average wavelet coefficient. The estimated frequency reflects the number of steps a person performs within this time window. Finally, the total number of steps was calculated as a sum of all 1-second step counts calculated over the duration of the observed period of walking.

The step-counting method described above is embedded into the walking recognition algorithm published in the public domain [31,32].

### Data Description

#### Overview

We evaluated the step-counting method in 3 ways, where each approach was selected to assess a different aspect of step-counting performance: (1) the cross-body validation aimed to determine the consistency of step counts across different body locations; (2) the visually assessed validation aimed to assess the method’s accuracy against step counts assessed visually by an observer; and (3) the commercial wearable validation aimed to assess the method’s step count compared with step counts obtained from a commercial, consumer-grade activity tracker (Fitbit Charge 2) worn at the wrist. Cumulatively, the entire validation was conducted using 8 independent data sets, including 7 data sets available in the public domain and 1 data set collected by our research team. All data sets are described in the following subsections.

#### Cross-Body Validation

For the cross-body validation, we identified 5 publicly available data sets, including Daily Life Activities (DaLiAc) [33], Physical Activity Recognition Using Smartphone Sensors (PARUSS) [34], RealWorld [35], Simulated Falls and Daily Living Activities (SFDLA) [36], and Human Physical Activity (SPADES) [37]. The data sets contained accelerometer data on walking activity collected simultaneously at several body locations that are representative of the everyday use of smartphones.

The aggregated cross-body validation data set included measurements collected from 103 healthy adults (Table 1) who performed walking activities in controlled environments (ie, all participants followed some predefined path), typically around a university campus (Table 2). One data set, RealWorld, involved participants walking outside in a parking lot and a forest.

Accelerometer data were collected using various wearable devices, including Android-based smartphones and research-grade wearable accelerometers from SHIMMER, Xsens Technologies, and ActiGraph. The devices were positioned at various locations across the body, that is, around the thigh, at the waist, on the chest, and on the arm (Table 3). Data set measurements differed based on data collection parameters, including the sampling frequency (eg, between 25 Hz in SFDLA and 204.8 Hz in DaLiAc) and measurement range (between ±6 g in DaLiAc and ±12 g in SFDLA). The measurement range was not provided in the PARUSS and RealWorld data sets.

**Table 1:** Demographics, body measures, and health status of participants involved in the data sets included in this study.

Validation scheme and data set			Participants, n		Male, n (%)		Age (years)				Height (cm)					Weight (kg)					BMI (kg/m^2^)					Health status
							Range		Mean (SD)		Range		Mean (SD)		Range			Mean (SD)		Range			Mean (SD)			
**Cross-body**																										
	DaLiAc^a^	19		11 (58)		18-55		26.5 (7.7)		158-196		177.0 (11.1)		54-108			75.2 (14.2)		17-34			23.9 (3.7)		Healthy		
	PARUSS^b^	10		10 (100)		25-30		N/A^c^		N/A		N/A		N/A			N/A		N/A			N/A		Healthy		
	RealWorld	15		8 (53)		16-62		31.9 (12.4)		163-183		173.1 (6.9)		48-95			74.1 (13.8)		18-35			24.7 (4.4)		Healthy		
	SFDLA^d^	17		10 (59)		19-27		21.9 (2.0)		157-184		171.6 (7.8)		47-92			65.0 (13.9)		17-31			21.9 (3.7)		Healthy		
	SPADES^e^	42		27 (64)		18-30		23.5 (3.1)		151-180		174.2 (8.5)		51-112			73.8 (15.0)		18-35			24.7 (4.1)		Healthy		
**Visually assessed**																										
	WalkRec^f^	77		N/A		N/A		N/A		N/A		N/A		N/A			N/A		N/A			N/A		Healthy		
	PedEval^g^	30		15 (50)		19-27		21.9 (52.4)		152-193		171.0 (10.8)		43-136			70.5 (17.6)		17-37			23.8 (3.7)		Healthy		
**Commercial wearable**																										
	HOPE^h^	45		0 (0)		24-79		61.5 (11.8)		148-172		159.9 (6.1)		48-107			67.8 (13.0)		19-43			26.5 (4.9)		Patients with advanced cancer		

^a^DaLiAc: Daily Life Activities.

^b^PARUSS: Physical Activity Recognition Using Smartphone Sensors.

^c^N/A: not applicable.

^d^SFDLA: Simulated Falls and Daily Living Activities.

^e^SPADES: Human Physical Activity.

^f^WalkRec: Walking Recognition.

^g^PedEval: Pedometer Evaluation Project.

^h^HOPE: Helping Our Patients Excel.

**Table 2:** Walking conditions in the data sets included in this study.

Validation scheme and data set			Measurement conditions		Activity description
**Cross-body**					
	DaLiAc^a^	Controlled		University campus	
	PARUSS^b^	Controlled		University building	
	RealWorld	Controlled		Paved (parking lot) and unpaved (forest) surfaces outdoors	
	SFDLA^c^	Controlled		University building	
	SPADES^d^	Controlled		University building	
**Visually assessed**					
	WalkRec^e^	Free-living		Natural conditions, freely or following some basic premises	
	PedEval^f^	Controlled and semicontrolled		2 laps around a designated gym path at a normal walking pace (controlled) Scavenger hunt: locating 4 objects in 4 rooms throughout a building (semicontrolled) Building a small Lego toy by assembling pieces distributed among 12 bins around a room and pattern-simulatedly preparing a meal in a kitchen (semicontrolled)	
**Commercial wearable**					
	HOPE^g^	Free-living		Natural conditions	

^a^DaLiAc: Daily Life Activities.

^b^PARUSS: Physical Activity Recognition Using Smartphone Sensors.

^c^SFDLA: Simulated Falls and Daily Living Activities.

^d^SPADES: Human Physical Activity.

^e^WalkRec: Walking Recognition.

^f^PedEval: Pedometer Evaluation Project.

^g^HOPE: Helping Our Patients Excel.

**Table 3:** Measurement parameters for the data sets included in this study.

Validation scheme and data set, and sensing device			Sensor location	Measurement range (g)	Sampling frequency (Hz)
**Cross-body**					
	**DaLiAc^a^**				
		Wearable accelerometer: SHIMMER	Waist and chest	±6	204.8
	**PARUSS^b^**				
		Smartphone: Samsung Galaxy S2	Thigh, waist, and arm	N/A^c^	50
	**RealWorld**				
		Smartphone: Samsung Galaxy S4	Thigh, waist, chest, and arm	N/A	50
	**SFDLA^d^**				
		Wearable accelerometer: Xsens MTw	Thigh, waist, and chest	±12	25
	**SPADES^e^**				
		Wearable accelerometer: ActiGraph GT9X	Thigh and waist	±8	80
**Visually assessed**					
	**WalkRec^f^**				
		Smartphone: BQ Aquaris M5	Unspecified	N/A	100
	**PedEval^g^**				
		Wearable accelerometer: SHIMMER3	Waist	±4	15
**Commercial wearable**					
	**HOPE^h^**				
		Smartphone: various Android- and iOS-based	Unspecified	Various	Various

^a^DaLiAc: Daily Life Activities.

^b^PARUSS: Physical Activity Recognition Using Smartphone Sensors.

^c^N/A: not applicable.

^d^SFDLA: Simulated Falls and Daily Living Activities.

^e^SPADES: Human Physical Activity.

^f^WalkRec: Walking Recognition.

^g^PedEval: Pedometer Evaluation Project.

^h^HOPE: Helping Our Patients Excel.

#### Visually Assessed Validation

Visually assessed validation was performed using 2 publicly available data sets: Walking Recognition (WalkRec) [38] and the Pedometer Evaluation Project (PedEval) [39]. The aggregated data set consisted of both raw accelerometer data for 107 healthy participants and ground truth step counts for each walking activity performed by study participants.

In this approach, walking activities were performed in controlled, semicontrolled, or free-living conditions. Specifically, WalkRec data set participants walked in settings of their choice without specific instructions (eg, indoor and outdoor walking along flat surfaces and climbing stairs; free-living), while PedEval data set participants performed three prescribed walking tasks: (1) a 2-lap stroll along a designated path (controlled), (2) a scavenger hunt across 4 rooms (semicontrolled), and (3) a toy-assembling assignment using pieces distributed across a dozen bins located around a room (semicontrolled). In the PedEval data set*,* step counts were visually assessed and manually annotated by a research team member, while in the WalkRec data set, the ground truth annotation was further augmented by recordings from a separate smartphone placed on each participant’s ankle.

The visually assessed validation data set was collected either by Android-based smartphones or a wearable accelerometer (SHIMMER3) placed around the waist (PedEval) or at various unspecified locations across the body (WalkRec). Each data set was collected with a different sampling frequency (WalkRec 15 Hz and PedEval 100 Hz), and only PedEval provided a measurement range (±4 g).

#### Commercial Wearable Validation

The commercial wearable validation data set was collected from patients with advanced gynecologic cancers receiving outpatient chemotherapy as part of the Helping Our Patients Excel (HOPE) study. The HOPE study aimed to assess the feasibility, acceptability, and perceived effectiveness of a mobile health intervention that used commercial wearable activity trackers and Beiwe, a digital phenotyping research platform, to collect accelerometer data, smartphone sensor data, and patient-reported outcomes. Patients were recruited from the outpatient gynecological oncology clinic at the Dana-Farber Cancer Institute in Boston, MA. The inclusion and exclusion criteria for study participation are described elsewhere [40].

The data set included 45 female patients with recurrent gynecologic cancers, including ovarian (n=34), uterine (n=5), cervical (n=5), and vulvar (n=1) cancers. Patients were asked to wear the Fitbit Charge 2 (Fitbit) on their nondominant wrist during all waking hours for a period of 6 months in a free-living setting. Each Fitbit was linked to the Fitabase analytics system (Small Steps Laboratories), which enabled the investigators to remotely monitor and export several metrics of patients’ physical activity, including minute-level step counts.

At baseline, patients were also asked to install Beiwe, the front-end component of the open-source, high-throughput digital phenotyping platform designed and maintained by members of the Harvard T.H. Chan School of Public Health [41]. Among other passive data streams, Beiwe collected raw accelerometer data with the default sampling rate (typically 10 Hz on most phones, which is sufficient for step counting) using a sampling scheme that alternated between on-cycles and off-cycles, corresponding to time intervals when the sensor actively collected data and was dormant, respectively. The smartphone’s accelerometer was configured to follow a 10-second on-cycle and a 20-second off-cycle. The sample scheme was identical on all participants’ smartphones.

### Data Preprocessing

Because each data set had different data collection parameters, we preprocessed the data sets to standardize the inputs in our algorithm. First, we verified if the acceleration data were provided in gravitational units (g); data provided in SI units were converted using the standard definition: 1 g = 9.80665 m/s^2^. Second, we used linear interpolation to impose a uniform sampling frequency of 10 Hz across triaxial accelerometer data. Third, we transformed the triaxial accelerometer signals into sensor orientation-invariant vector magnitudes.

### Statistical Analysis

The available accelerometer data were processed using the walking recognition and step-counting algorithm with default tuning parameters, as previously described [30]. Depending on the validation approach, the resulting 1-second step counts were then aggregated into step counts for the entire walking bout or specified time fragment. For the cross-body and visually assessed validations, step counts were calculated as a sum of all step counts in each walking bout and for each sensor location.

Additional analyses were required for commercial wearable validation. Here, step counts were first aggregated on a minute level, the smallest time resolution available to export from Fitabase. Because the Beiwe sampling scheme follows on and off cycles, we adjusted the observed smartphone-based step counts by a proportional recovery based on the ratio between the duration of data collection (20 seconds) and noncollection (40 seconds) in each 1-minute window by multiplying them by 3. Further, due to a lack of information on both wearable and smartphone wear-time and a potential time lag between measurements between the 2 devices, we removed minutes with 0 steps recorded by either method. Finally, to allow for a direct comparison, we summed the smartphone-based step counts for each day of observation.

Each validation procedure considered a different ground truth step count for comparison. In the cross-body validation sample, we compared step counts estimated from various body locations for the entire walking bout. For example, if the data set included data from 3 sensors located on the thigh, waist, and arm, we would compare step counts between the thigh and waist, thigh and arm, and waist and arm. In the visually assessed validation sample, we compared step counts estimated from the available sensor location to a visually assessed ground truth for the entire walking bout. In the commercial wearable validation sample, we compared the daily number of steps estimated from the smartphone to step counts provided by Fitbit. This procedure was performed using 2 days of observations for each patient. The first day was identified as the first full day of observations for each patient. Given that some patients recorded very few steps on that day (possibly due to limited wear time), we also compared step counts from the first day and a day with at least 1000 observed steps on the smartphone to allow for a more in-depth assessment of the algorithm. For a more detailed evaluation, we conducted an additional analysis on minute-level data collected during the first day of observation.

We created Bland-Altman plots for each data set, and all of the data sets were combined within each validation scheme. Mean bias and limits of agreement (LoA) were calculated to describe the level of agreement between step counts. The mean bias was calculated as the mean difference between 2 methods of measurement, while LoA were calculated as the mean difference ±1.96 SD. Participant demographics, body measures, and step count statistics were reported as a range and mean (SD), whenever available.

In addition, we evaluated our method for algorithmic fairness to demographic and anthropometric descriptors. Specifically, we fitted 3 linear regression models into the data set collected for commercial wearable validation. The first model was specified as *Y_i_* = *β*_0_+*βX_i_*+*ε_i_*, where *Y_i_* is the difference between the step counts from the smartwatch and smartphone collected during the first day of observation for participant *i*, *β* is the vector of coefficients for the covariates, *X_i_* is the vector of covariates (age and BMI), and *ε_i_* is random noise. The second model was similar to the first, but *Y_i_* is now the difference between step counts from the smartwatch and smartphone collected during the first day of observation for participants with at least 1000 observed smartphone steps. The third model used a linear mixed-effects regression analysis to account for the clustering of the data within participants. The model was specified as *Y_i_ = β*_0_+*βX_i_*+*b_i_*+*ε_i_*. In contrast to the first and second models, here we include a random intercept *b_i_* for each participant *i*. In each analysis, we calculated 95% CI to assess statistical significance.

Step counts were calculated in Python using a previously published open-source method [32]. Statistical analysis and visualization were prepared in MATLAB (R2022a; MathWorks).

### Ethical Considerations

The HOPE study was approved by the Dana-Farber/Harvard Cancer Center institutional review board (protocol 16-477).

## Results

### Cross-Body Validation

The aggregated cross-body validation data set consisted of data from healthy 103 participants (66 males, representing 64% of the data set) between 16 and 62 (mean 25.2, SD 7.1) years of age. All data sets, except for PARUSS, provided data on participants’ height and weight, which ranged between 151 and 196 (mean 173.8, SD 8.5) cm and 47 and 112 (mean 72.2, SD 14.7) kg, respectively. Participants’ BMI ranged between 17 and 35 (mean 23.8, SD 4.1) kg/m^2^.

In this validation, step counts were aggregated separately for each walking bout across different body locations, including the thigh (n=83 bouts), waist (n=102), chest (n=51), and arm (n=25). Cumulatively, we examined 232 sensor body location pairs: thigh versus waist (n=83), thigh versus chest (n=32), thigh versus arm (n=25), waist versus chest (n=51), waist versus arm (n=25), and chest versus arm (n=15).

On average, in the aggregated cross-body validation data set, participants performed a mean of 751.7 (SD 581.2) steps per walking bout. Mean step counts varied by the data set (participants’ mean step counts were 501.5, SD 127.2 in DaLiAc; 337.5, SD 14.6 steps in PARUSS; 1007.2, SD 79.6 steps in RealWorld; 14.6, SD 1.7 steps in SFDLA; and 1408.7, SD 561.5 steps in SPADES).

**Figure 2:**
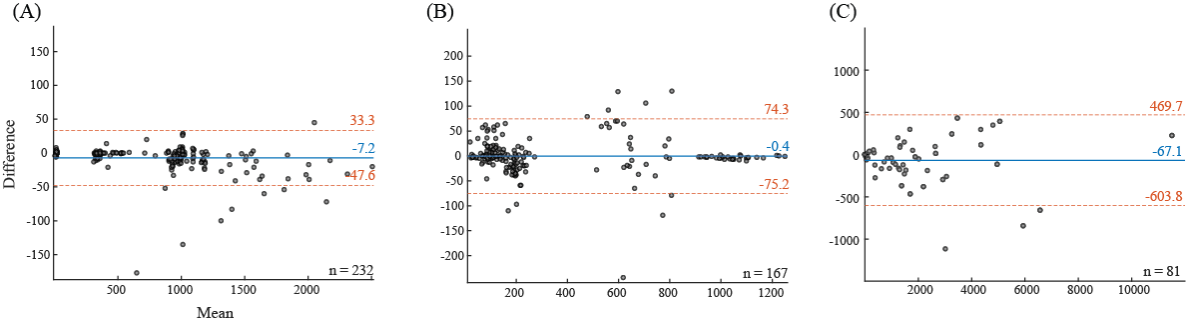
Bland-Altman plots with comparison of step counts in 3 validation approaches: (A) internal, (B) manual, and (C) wearable. (A) The horizontal axis indicates a mean step count from the 2 body locations; (B) estimated steps and manually counted ground truth; and (C) estimated steps and step counts obtained from Fitbit. The vertical axis indicates a difference between step counts from the 2 methods. Blue solid lines indicate mean bias, while dashed red lines indicate ±95% limits of agreement calculated as ±1.96 SD of the differences between the 2 methods.

Figure 2A displays the Bland-Altman plots for the aggregated cross-body validation data set. Comparisons between individual studies are provided in Figures A-E in Multimedia Appendix 1. Across the aggregated data set, the mean bias was equal to –7.2 (LoA –47.6, 33.3) steps, or –0.5%. The largest relative overestimation observed was between the waist and chest in the SFDLA data set and equaled 1.2 (LoA –4.3, 6.8) steps, or 8.5% of the total steps. The largest underestimation was observed between the thigh and waist in the SPADES data set and equaled –28.7 (LoA –107.1, 49.7) steps, or –2.0% of the total steps.

### Visually Assessed Validation

The visually assessed validation of our method included 107 healthy participants. Demographic and anthropometric measurements were only available in the PedEval data set. This data set combined 30 participants, 15 of whom were males, whose ages ranged between 19 and 27 (mean 21.9, SD 52.4) years, whose heights ranged between 152 and 193 (mean 171.0, SD 10.8) cm, and whose weights ranged between 43 and 136 (mean 70.5, SD 17.6) kg. Participants’ BMIs ranged between 17 and 37 (mean 23.8, SD 3.7) kg/m^2^.

We estimated the step count bias based on 167 comparisons, including 77 comparisons from the WalkRec data set and 90 from the PedEval data set (30 per task). Participants’ mean step count in the aggregated visually assessed validation data set was 367.4 (SD 359.4) steps according to the ground truth (Figure 2B). WalkRec data set participants’ mean steps were 126.8 (SD 59.2) steps, while PedEval data set participants’ steps varied by activity and were 1025.0 (SD 171.3) steps in task 1; 648.5 (SD 126.3) steps in task 2; and 179.2 (SD 22.7) steps in task 3 (Figures F-G in Multimedia Appendix 1). The corresponding estimations calculated using our method were a mean of 119.8 (SD 62.2) steps for the WalkRec data set; 1027.5 (SD 175.0) steps for task 1; 641.1 (SD 137.3) steps for task 2; and 210.8 (SD 18.7) steps for task 3. The mean bias across the aggregated data set was –0.4 (LoA –75.2, 74.3) steps, or 0.1%. The largest relative overestimation was +8.8 (LoA –32.1, 49.7) steps, or 6.9%, within the WalkRec data set. The largest underestimation was –32.3 (LoA, –80.4, 15.8) steps, or –18%, observed in task 3 in the PedEval data set.

### Commercial Wearable Validation

Our commercial wearable validation included data from 45 female patients with advanced gynecological cancers. Their ages ranged between 24 and 79 (mean 61.5, SD 11.8) years. Their heights ranged between 148 and 172 (mean 159.9, SD 6.1) cm, weights ranged between 48 and 107 (mean 67.8, SD 13.0) kg, and BMIs ranged between 19 and 43 (mean 23.8, SD 3.7) kg/m^2^.

Our Bland-Altman analysis included over 81 observations of daily step counts (Figure 1C), involving 45 days that constituted the first full day of observation (Figure G in Multimedia Appendix 1) and 36 first days with at least 1000 steps estimated from a smartphone (Figure H in Multimedia Appendix 1). A total of 9 participants did not have any days with more than 1000 steps observed, likely due to limited smartphone wear-time. In the aggregated data set, the algorithm estimated a mean daily step count of 1998.2 (SD 2350.3) steps, which included a mean daily step count of 1371.3 (SD 2343.1) steps observed during the first day and 2816.7 (SD 2123.6) steps during the first day with at least 1000 steps observed. Comparisons with data from Fitbit were similar, including a mean daily step count of 1931.2 (SD 2338.4) across participants, a mean daily step count of 1316.4 (SD 2320.2) steps during the first day, and a mean daily step count of 2733.7 (SD 2136.9) steps during the first day with at least 1000 steps observed, respectively. The aggregated estimation bias of the smartphone versus the Fitbit was –67.1 (LoA –603.8, 469.7) steps, or 3.4%, with an underestimation of –54.9 (LoA –485.3, 375.6) steps, or –4.2%, during the first day, and –83.0 (LoA –738.5, 572.6) steps, or –3.0%, during the first day with at least 1000 steps.

Further analysis showed that mean minute-level step counts from Fitbit and smartphone were equal to 51.4 (SD 37.1) and 53.5 (SD 34.3) steps, respectively, which underlines a close alignment between the 2 approaches. Additionally, Bland-Altman analysis (Multimedia Appendix 2) revealed that the estimation bias was equal to –2.1 (LoA –41.6, 37.3) steps and suggested that the smartphone algorithm predominantly overcounted steps in minutes with a few to several steps taken and undercounts steps in minutes with 100 steps and more. Unfortunately, due to the free-living nature of observation, we were unable to determine which activities are especially prone to overcounting steps, yet we hypothesize that it might occur during household activities that require taking a few steps at a time, preceded or followed by body rotations, such as preparing a meal or cleaning. The discrepancies might also result from the potential time lag between measurements.

The evaluation of algorithm fairness revealed no systematic bias for any included covariate (Table 4).

**Table 4:** Step-counting bias estimation in the commercial wearable validation data set.

Modeling approach and covariates		Estimate	SE	95% CI
**First day**				
	Intercept	–90.6	258.8	–612.8 to 431.6
	Age	–1.4	2.9	–7.3 to 4.5
	BMI	4.5	7.0	–9.7 to 18.6
**First day with** **≥** **1000 steps**				
	Intercept	59.3	461.4	–879.4 to 998.0
	Age	–1.3	4.7	–10.8 to 8.2
	BMI	–2.4	14.2	–31.2 to 26.5
**Both**				
	Intercept	–39.2	283.8	–604.2 to 525.9
	Age	–1.3	3.2	–7.6 to 5.0
	BMI	2.1	7.8	–13.5 to 17.6

## Discussion

In this study, we conducted a 3-way validation of the open-source step-counting method for smartphone data and demonstrated that it provides reliable estimates across various sensor locations, measurement conditions, and populations. The validation was carried out using a previously published walking recognition method for body-worn devices that contain an accelerometer [30]. This method leverages the observation that regardless of sensor location on the body, during walking activity, the predominant component of the accelerometer signal transformed to the frequency domain, that is, step frequency, remains the same, enabling the calculation of the number of steps a person performed in a given time fragment. In our previous study, we validated this approach for walking recognition using data from 1240 participants gathered in 20 publicly available data sets, and demonstrated that our method estimates walking periods with high sensitivity and specificity: the average sensitivity ranged between 0.92 and 0.97 across various body locations, and the average specificity was largely above 0.95 for common daily activities (household activities, using motorized transportation, cycling, running, desk work, sedentary periods, eating, and drinking). Importantly, the method’s performance was not sensitive to different demographics and metrological factors for individual participants or studies, including participants’ ages, sexes, heights, weights, BMIs, sensor body locations, and measurement environments.

In this study, we further extend this work by validating the performance of the step-counting method using data collected from 255 participants in 8 independent studies with three goals in mind: (1) assessment of the concordance of step counts across various body locations, (2) comparison of the method’s estimates with visually observed step counts, and (3) comparison of the method’s estimates with indications of commercial activity tracker (Fitbit Charge 2). The first comparison, a cross-body validation, demonstrated very high agreement between step counts measured from smartphones located at most of the places where smartphones are typically worn, that is, the thigh, waist, chest, and arm. This result suggests that our method can be used to assess steps without restricting where participants wear their smartphones, which may reduce participant burden during data collection and help improve long-term study adherence.

Our visually assessed validation of uninterrupted walking revealed almost perfect agreement between the step counts estimated with our method and those denoted by a visual observer. In this case, the absolute difference observed between the 2 measures was consistently below 1% (Figure G in Multimedia Appendix 1), which is similar to the results achieved with deep learning methods validated on this data set in the past [42,43]. These results reinforce the utility of using this method in controlled conditions, for example, to evaluate participants’ functional capacity using a 6-minute walk test, and indicate that the method provides highly accurate estimation of step counts across various sensor locations during regular flat walking.

The mean step-counting bias was also low for semicontrolled walking tasks recorded in the PedEval data set, free-living tasks recorded in the WalkRec data set, and for both scenarios within the commercial wearable validation (first day and first day with at least 1000 steps). In these instances, however, the analysis revealed a wider LoA, which may result from a more complex structure of the underlying data, which involved walking only a few steps at a time as well as sudden changes in walking direction and altitude (eg, stair climbing) [44]. As discussed previously [30], in walking signals with such characteristics, the step frequency tends to be modulated by its sub- and higher harmonics, which might be identified as dominant in the wavelet decomposition outcome and mislead our method.

Even more challenging data were analyzed in the commercial wearable validation cohort. Here, the data were collected at unspecified locations (including novel locations, eg, a bag or backpack) and included data representing various activities of daily living, such as grocery shopping, riding in a car, and doing dishes, which might artificially inflate the estimated step counts by either method. This is a likely reason why the comparisons had wider discrepancies, even after removing minutes with 0 steps recorded by either device. Nevertheless, the estimated bias remained low, which indicates that our validated method provides reliable step count estimates across populations and conditions.

Our analysis has several limitations that should be addressed in future studies. First, due to the lack of available data sets, our method was not validated in individuals with walking impairments or those requiring walking aids, such as cane or walkers. Similarly, this method has not been validated in children and many elders, although the mean age of participants in our commercial wearable validation set was 61.5 years, and over 11% (5/45) were 74 years of age or older. Further research is needed to understand the frequency-domain gait characteristics in the presence of limping, as well as the potential overlap between the step frequency of walking activity in children and that of running activity in adults [45,46]. The latter might be particularly important in studies that differentiate steps performed during leisure and exertional activities. Second, commercial wearable validation was performed with the use of a proprietary activity tracker (Fitbit Charge 2). Although this device has demonstrated reliable step counts during naturalistic gait performed in laboratory conditions [47,48], its accuracy in free-living conditions is inconclusive, and it is presumably dependent on the characteristics of the studied population [21,49,50]. Importantly, the selected activity tracker was placed on the wrist, a body location that can be activated by many repetitive movements (eg, gesticulating) while the rest of the body is still; hence, it is more likely to overestimate steps compared to locations closer to the body mass center. To improve comparisons with our method, in commercial wearable validation, we removed data instances when either method indicated 0 steps. Finally, the estimation of step counts in free-living studies must account for nonwear time of smartphones (eg, while the phone is charging or sitting on a table). Unlike many wearables that are attached to the body (eg, wristbands), smartphones can be easily set aside, sometimes for prolonged periods of time. Such situations introduce a considerable discrepancy between the estimated and actual number of steps a person performs during the day and should be reported, ideally with CIs. Future research should also consider systematic identification, estimation, and imputation of step counts during periods when the sensor is not being worn.

In conclusion, we performed a 3-way validation of a robust, reproducible, and scalable method for step counting using smartphones and other wearable activity trackers. This validation demonstrates that our approach provides reliable step counts across sensor locations and populations, including healthy adults and those with incurable cancers. The method performed well in multiple environments, including indoors, outdoors, and in day-to-day life across settings. This method is a promising strategy for studying human gait with personal smartphones that does not require active patient participation or the introduction of new devices.

## Appendix

Multimedia Appendix 1 Bland-Altman plots with comparison between step estimates in the included studies: (A) DaLiAc, (B) PARUSS, (C) RealWorld, (D) SFDLA, (E) SPADES, (F) WalkRec, (G) PedEval, and (H) HOPE. In PedEval, * indicates task 1, ** indicates task 2, and *** indicates task 3. In HOPE, † indicates analysis over the first full day past enrollment and †† indicates analysis over the first full day with more than 1000 steps. Algorithm performance was compared using step estimates from various body locations (A - E), manual annotation (F - G), and Fitbit (H).

Multimedia Appendix 2 Bland-Altman plots with comparison of minute-level step counts from wearable validation. The horizontal axis indicates a mean step count between estimated steps and step counts obtained from Fitbit. The vertical axis indicates a difference between step counts from the two methods. Blue solid lines indicate mean bias while dashed red lines indicate ±95% limits of agreement calculated as ±1.96 of standard deviations of the differences between the two methods.

## Abbreviations

DaLiAc: Daily Life Activities
HOPE: Helping Our Patients Excel
LoA: limits of agreement
PARUSS: Physical Activity Recognition Using Smartphone Sensors
PedEval: Pedometer Evaluation Project
SFDLA: Simulated Falls and Daily Living Activities
SPADES: Human Physical Activity
WalkRec: Walking Recognition
