# Supplementary figures and images for: Open-Source, Step-Counting Algorithm for Smartphone Data Collected in Clinical and Nonclinical Settings: Algorithm Development and Validation Study
Source: JMIR Cancer. 2023 Nov 15;9:e47646. doi: 10.2196/47646 (PMC10687676; doi:10.2196/47646)

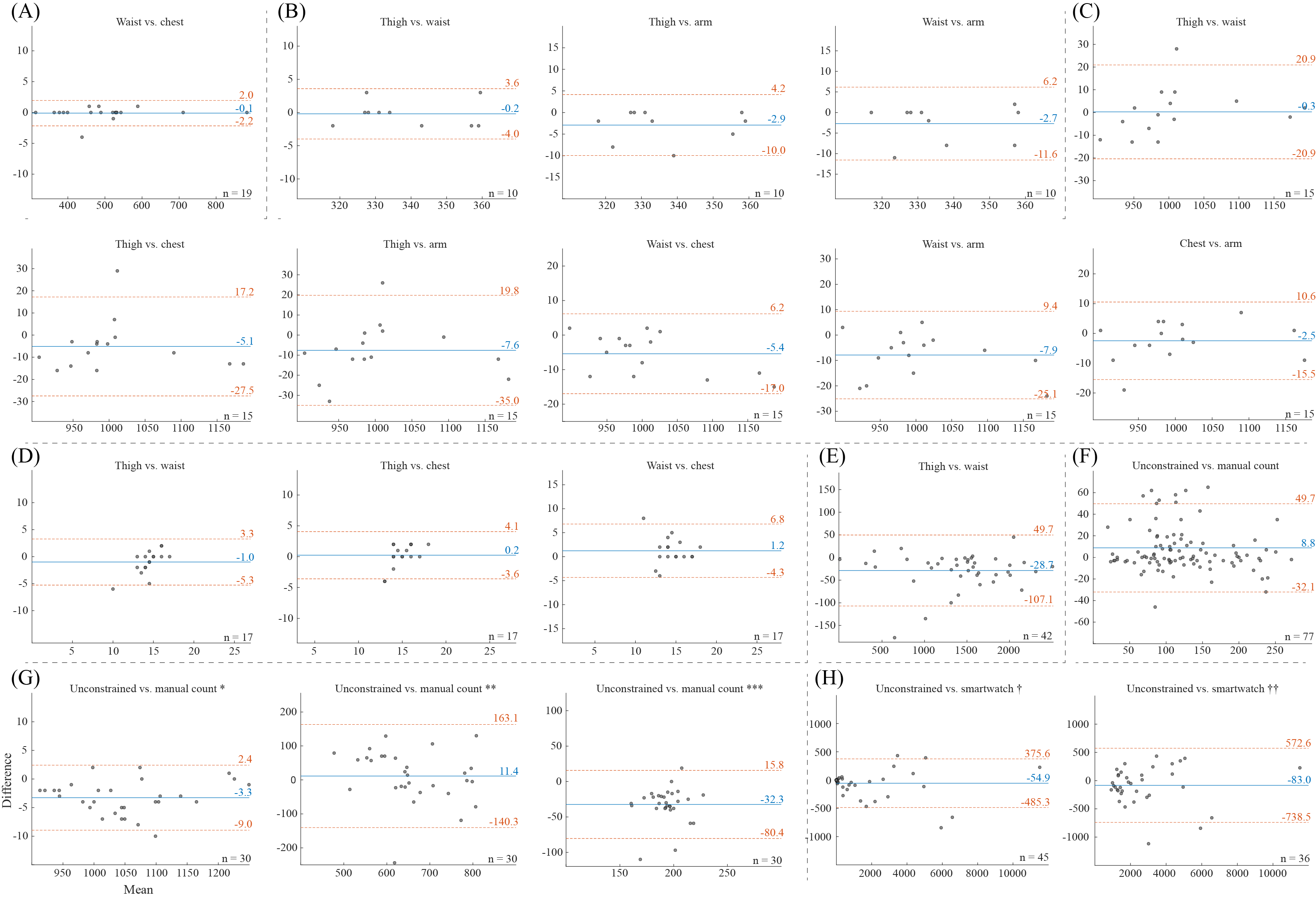

Supplement: Multimedia Appendix 1 [file cancer_v9i1e47646_app1.png]

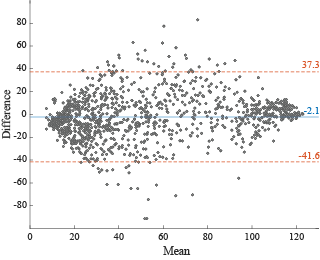

Supplement: Multimedia Appendix 2 [file cancer_v9i1e47646_app2.png]
